# Association of serum neurofilament light chain with cognitive impairment: findings from the National Health and Nutrition Examination Survey

**DOI:** 10.3389/fnagi.2025.1517663

**Published:** 2025-01-20

**Authors:** Tianjiao Meng, Qinwen Fei, Tian Lv, Shiqin Chen

**Affiliations:** ^1^Department of Neurology, Zhuji Affiliated Hospital of Wenzhou Medical University, Zhuji, China; ^2^Department of Geriatrics, Zhuji Affiliated Hospital of Wenzhou Medical University, Zhuji, China; ^3^Department of Neurology, Second People's Hospital of Yuhuan, Yuhuan, China

**Keywords:** cognitive function, serum neurofilament light chain, RCS, nomogram, NHANES

## Abstract

**Background:**

Serum Neurofilament Light chain (NfL) is a promising biomarker of neuronal damage, used to assess the extent of neuronal injury and neurodegeneration, and it is widely applied in the diagnosis of neurodegenerative disease and monitoring disease progression. This article aims to determine whether serum NfL associated with cognitive level.

**Methods:**

Using NHANES data, we conducted an analysis of cognitive test results for 450 adults aged 60 years and older and examined their correlation with serum NfL levels. When exploring the association between cognitive test scores and serum NfL levels, regression models and restricted cubic spline (RCS) regression models were employed to adjust for potential confounding factors. The least absolute shrinkage and selection operator (LASSO) regression was applied for identifying key cognitive impairment factors, which was then included in the establishment of a risk prediction nomogram model, with the receiver operating characteristic (ROC) curve being built to evaluate its discriminatory power for cognitive impairment.

**Results:**

It was found that there is a strong positive correlation between serum NfL levels and both low total cognitive function (total-CF) OR: 1.028 (95%CI = 1.015–1.041 *p* < 0.001) and low Digit Symbol Substitution Test (DSST) OR: 1.026 (95%CI = 1.003–1.050, *p* = 0.027). Furthermore, using the RCS model, we observed a linear trend in the relationship between NfL and low total-CF. The nomogram model based on NfL identified by LASSO regression displayed a considerable predicative value for low total-CF, with an area under the curve [AUC = 85.6% (81.6–89.3%)].

**Conclusion:**

There is a strong correlation between serum NfL levels and cognitive function, especially DSST, which reflects attention and information processing abilities, as well as overall cognitive function, but not memory and language fluency. Thus, NfL may serve as a serum biomarker for dementia monitoring.

## Introduction

Cognitive impairment is a pressing global health concern, with millions of people worldwide estimated to suffer from dementia (GBD 2019 Dementia Forecasting Collaborators, 2022). As the global population continues to age, the prevalence of cognitive impairment is on an upward trajectory. Global epidemiological studies show that the prevalence of dementia seems to double every five years for elderly individuals aged between 50 and 80 (Palmer et al., 2022), with a more pronounced impact in developing countries. This condition can stem from various causes, including neurodegenerative diseases such as Alzheimer’s disease.

In clinical practice, when confronted with cognitive decline arising from nonneurological factors such as psychosocial influences, anemia, malnutrition, sleep disturbances, cardiovascular diseases, and others, physicians must undertake a comprehensive assessment to ascertain the specific underlying causes (Kung et al., 2021; Piolatto et al., 2022). At this juncture, the early detection and intervention in cognitive function hold the potential to ameliorate clinical symptoms and enhance the quality of life for affected patients.

Neurofilaments (Nfs) constitute the principal components of the axonal cytoskeleton, comprising three subunits: the NF light chain (NfL), intermediate chain, and heavy chain, with exclusive expression in neurons. Among these subunits, NfL plays a pivotal role in maintaining the structural integrity of neurons. Typically, under normal conditions, NfL is released at low levels from axons as individuals age, resulting in elevated NfL levels in the elderly (Gaetani et al., 2019; Khalil et al., 2020). Cross-sectional studies have revealed that elderly individuals with mild cognitive impairment (MCI) exhibit higher plasma NfL levels than cognitively normal elderly individuals. Longitudinal studies further suggest that individuals with higher baseline plasma NfL concentrations demonstrate a decline in cognitive function over time (Mattsson et al., 2017; Mielke et al., 2019). Whenever inflammation, neurodegeneration, trauma, or vascular injury occurs, leading to axonal damage within the nervous system, NfL is released into the interstitial fluid. The interstitial fluid is in direct communication with both blood and cerebrospinal fluid. Therefore, an elevation in NfL levels in the body can serve as a valuable biological biomarker indicative of neuronal injury (Yuan et al., 2017).

This study, based on the large-scale representative sample of NHANES, combined advanced statistical methods such as LASSO regression, for the assessed the potential application of NfL in cognitive impairments. By establishing an accurate risk prediction model, this study provides new tools for the early diagnosis of cognitive disorders and further promotes the translation of NfL into clinical applications.

## Materials and methods

### Study sample and design

The National Health and Nutrition Examination Survey (NHANES) is a complex, multistage, representative sample survey in the United States. It is conducted annually to systematically collect demographic and lifestyle information, aiming to observe the health and nutritional status of people living in the U.S. The survey consists of five main components: demographic data, laboratory tests, dietary information, physical examinations, and questionnaire responses. NHANES 2013–2014 is the only cycle that provides data on serum-detected NfL, offering a unique opportunity for this study.

**Figure 1:**
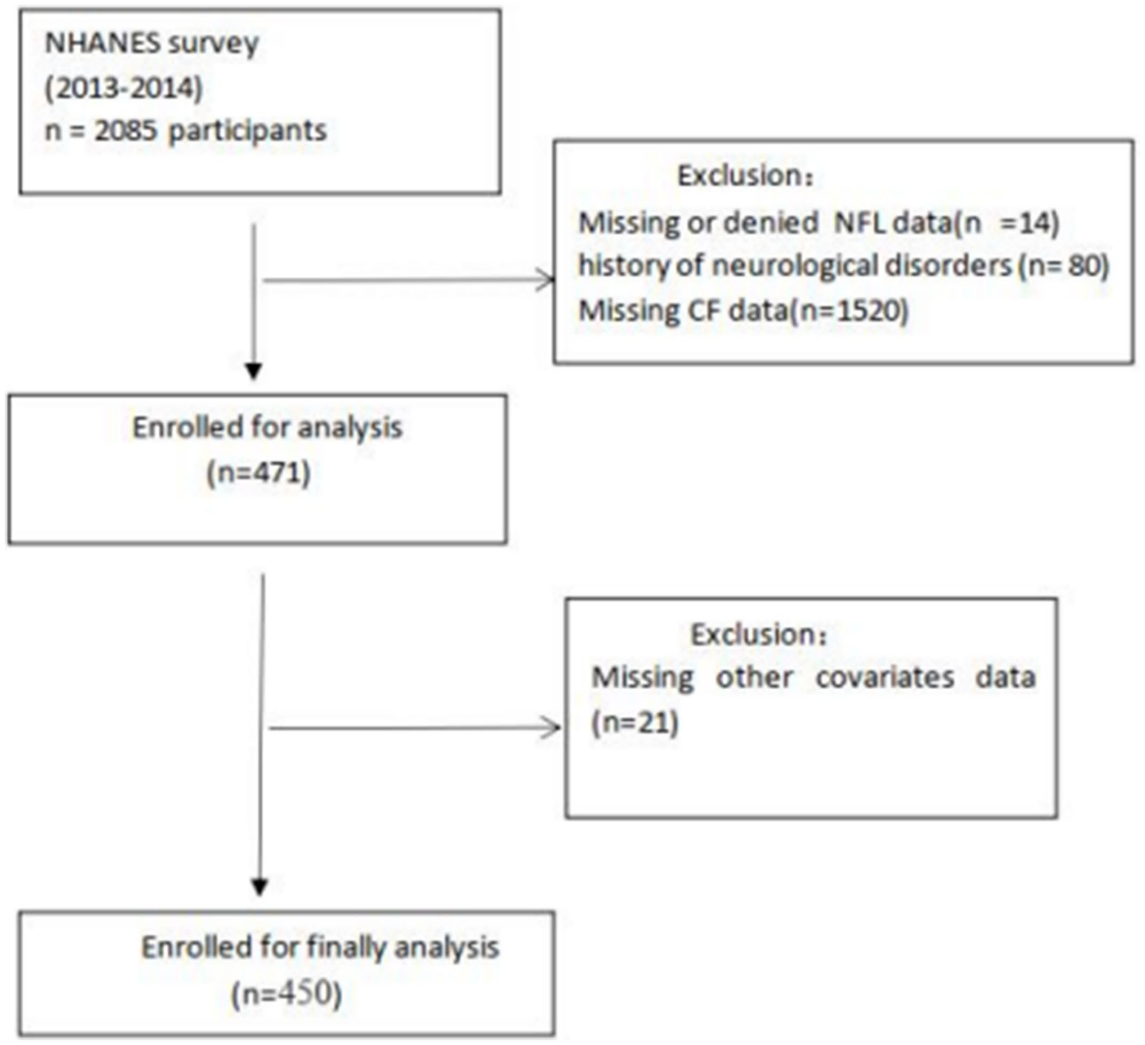
A detailed flow chart of participant recruitment.

In this study, we surveyed 2,085 subjects aged ≥60 years from the NHANES 2013–2014 cycle and assessed their cognitive performance. A total of 471 participants with valid cognitive function data were included, after excluding those with missing or declined NfL data, missing cognitive function data, or a history of neurological diseases. After excluding participants with missing data for education (*n* = 1), smoke (*n* = 1), alcohol statue (*n* = 8), BMI (*n* = 5), chronic kidney disease (*n* = 3), hemoglobin (*n* = 2), sleep duration (*n* = 1). Ultimately, 450 participants were included in the final analysis. The participant flow diagram is shown in Figure 1.

### Variables

The primary exposure variable in this study was NfL. In the NHANES database, NfL levels were measured using a highly sensitive immunoassay developed by Siemens Healthineers, which is based on acridinium ester (AE) chemiluminescence technology and paramagnetic particles. This assay runs on the high-throughput, automated Atellica platform, ensuring precision and reproducibility. The detailed protocol and additional information are available through NHANES.^1^

Outcome variable was cognitive function (CF). We assessed CF using three variables: the Consortium to Establish a Registry for Alzheimer’s Disease Word Learning subtest (CERAD W-L), Animal Fluency test (AFT), and Digit Symbol Substitution test (DSST).

The CERAD W-L evaluates immediate and delayed learning ability for new verbal information, specifically in the memory subdomain (Casagrande et al., 2021). Participants read a set of 10 unrelated words, followed by an immediate recall test (IRT) where they were asked to recall as many words as possible. The delayed recall test (DRT) occurred approximately 10 min later. Each trial’s maximum score is 10, with a total word list score of 40.

#### Animal Fluency Test (AFT)

The AFT assesses verbal fluency, an aspect of executive function (Clark et al., 2009), while also evaluating semantic memory and processing speed. Participants are tasked with listing as many animals as possible within one minute, receiving one point for each correctly named animal. The minimum score is 0, and the maximum score is 35 in our study population.

#### Digit Symbol Substitution Test (DSST)

The DSST is a module from the Wechsler Adult Intelligence Scale III that measures processing speed, sustained attention, and working memory. Participants are asked to copy as many corresponding symbols as possible into 133 boxes adjoining numbers within two minutes (Bienias et al., 2003; Golub et al., 2020). In our study population, the maximum score is 105, and the minimum score is 5.

#### Total Cognitive Function (Total-CF)

The Total-CF score is the sum of scores from DSST, AFT, and the Consortium to Establish a Registry for Alzheimer’s Disease Word Learning subtest (CERAD W-L). The Total-CF score ranges from 25 to 125 in our study population.

To define abnormal scores, we established cutoff values based on the lowest quartile within our study population, following methodologies employed in prior studies (Li et al., 2019) These cutoff points were applied to indicate low cognitive function across individual tests (IRT, DRT, DSST, AFT, CERAD W-L) and the Total-CF composite score.

Covariates were categorized into three groups: demographic data, medical conditions, and examination results. Demographic factors included age, gender, race, education, poverty-income ratio (PIR), smoking status (never, former, now), alcohol use (never, former, mild, moderate, heavy), and obesity (body mass index (BMI) < 30 kg/m^2^, BMI ≥ 30 kg/m^2^). Medical conditions included chronic kidney disease (CKD), hyperlipidemia, cancer, and hypertension. Examination results included albumin, Scr (serum creatinine), NLR (neutrophil-to-lymphocyte ratio), neutrophil count (neu), lymphocyte count (lym), bilirubin, protein, and uric acid.

### Statistical analysis

Our study considered complex sampling designs and sampling weights, utilizing NfL weights for all analyses (see Footnote 1). Baseline characteristics are presented as the means and standard errors (SE) for continuous variables and proportions for categorical variables. NfL was categorized into quartiles. Student’s t tests were employed for continuous variables, and chi-square tests were used for categorical variables to compare the NfL quartile groups.

A model was employed to estimate the odds ratio (OR) and 95% confidence intervals (CI) for the association between NfL and cognitive function. Criteria for selecting adjusted variables were determined through consideration of biological factors and a review of literature on the topic.

Our statistical inferences were based on three models: the crude model had no adjusted variables. Model 1 incorporated age, sex, education, and race. Model 2 included all variables from Model 1, as well as alcohol status, history of CKD, hypertension, and, PIR, PHQ-9 score.

This study stratified users based on age (individuals over 70, individuals under 70), sex (male, female), BMI (< 30, ≥ 30 kg/m^2^), smoking status (never, former, now), history of hyperlipidemia (yes, no), and history of hypertension (yes, no).

A stratification analysis explored the correlation between NFL and low total-CF. We employed the restricted cubic spline (RCS) regression model to flexibly model the association between NfL and low total-CF. We applied the least absolute shrinkage and selection operator (LASSO) regression model. In LASSO model, we used the method of cross-validation for model evaluation and parameter selection where the dataset is divided into 10 subsets, and the model is trained and tested on the 10 subsets multiple times. Besides, a risk prediction nomogram model was developed based on several key low-CF related variables, with its discriminatory power for forecasting the risk of low-CF being validated by the receiver operating characteristic (ROC) curve, A concordance index (C-index), calibration curves and a decision curve analysis (DCA) were used to measure the predictive performance of the nomogram.

Statistical analyses were performed using R Studio 4.2.0, with the level of statistical significance set at *p* < 0.05.

## Results

### Baseline characteristics

The data analyzed originated from 450 participants. NfL was categorized into quartiles: Q1 (< 14.1 pg./mL), Q2 (14.1–18.7 pg./mL), Q3 (18.7–26.78 pg./mL), and Q4 (≥ 26.78 pg./mL). Table 1 presents baseline characteristics according to the NfL quartiles.

**Table 1:** Descriptive baseline characteristics of participants.

	Total	Q1	Q2	Q3	Q4	*P* value
Age	66.261 (0.221)	64.415 (0.497)	66.161 (0.355)	66.931 (0.440)	67.514 (0.384)	< 0.001
Sex						0.714
Female	234 (52.493)	60 (50.726)	61 (57.450)	60 (52.595)	53 (49.139)	
Male	216 (47.507)	53 (49.274)	51 (42.550)	53 (47.405)	59 (50.861)	
Education						0.157
<High	111 (14.472)	23 (14.537)	25 (11.688)	34 (19.401)	29 (12.650)	
College	234 (65.438)	66 (75.976)	63 (66.378)	53 (59.529)	52 (59.847)	
High	105 (20.090)	24 (9.486)	24 (21.934)	26 (21.070)	31 (27.503)	
Race						0.325
Black	85 (7.187)	23 (8.125)	20 (6.397)	18 (6.481)	24 (7.728)	
Other	156 (13.417)	48 (15.856)	36 (11.686)	43 (17.394)	29 (9.145)	
White	209 (79.397)	42 (76.018)	56 (81.917)	52 (76.125)	59 (83.128)	
PIR	3.172 (0.154)	3.341 (0.244)	3.258 (0.190)	3.088 (0.248)	2.997 (0.282)	0.725
BMI (kg/m^2^)	29.139 (0.276)	30.012 (0.823)	28.724 (0.321)	27.964 (0.659)	29.801 (0.533)	0.13
Sleep duration (hour)	7.109 (0.099)	7.095 (0.107)	7.236 (0.087)	6.951 (0.178)	7.141 (0.201)	0.278
PHQ-9 score	3.566 (0.208)	3.278 (0.330)	3.162 (0.632)	2.607 (0.382)	5.129 (0.598)	0.034
Uric acid (mg/dl)	5.571 (0.114)	5.374 (0.136)	5.401 (0.136)	5.468 (0.187)	6.025 (0.278)	0.231
Protein(g/L)	69.425 (0.245)	69.034 (0.461)	69.058 (0.533)	69.777 (0.577)	69.844 (0.428)	0.51
Bilirubin (mg/dL)	0.659 (0.013)	0.659 (0.037)	0.684 (0.025)	0.645 (0.024)	0.648 (0.023)	0.527
Serum creatinine (mg/dL)	0.965 (0.039)	0.845 (0.021)	0.897 (0.031)	0.918 (0.023)	1.190 (0.164)	0.015
Neutrophil (%)	58.493 (0.634)	56.879 (1.094)	57.887 (1.076)	59.583 (1.576)	59.642 (1.110)	0.07
Lymphocyte (%)	28.643 (0.631)	29.944 (0.800)	28.876 (1.193)	27.973 (1.498)	27.780 (1.026)	0.193
NLR	2.379 (0.095)	2.170 (0.141)	2.331 (0.154)	2.513 (0.242)	2.503 (0.159)	0.225
IRT	21.483 (0.263)	22.105 (0.422)	21.806 (0.371)	21.276 (0.352)	20.755 (0.398)	0.073
DRT	7.023 (0.180)	7.192 (0.187)	7.392 (0.219)	7.036 (0.322)	6.479 (0.258)	0.012
CERAD W-L	28.506 (0.429)	29.297 (0.566)	29.199 (0.567)	28.312 (0.635)	27.234 (0.621)	0.033
DSST	54.840 (1.262)	58.477 (2.210)	56.522 (2.133)	54.822 (1.170)	49.692 (1.387)	0.003
AFT	18.814 (0.421)	20.223 (1.113)	19.801 (0.733)	17.907 (0.658)	17.315 (0.644)	0.014
Total-CF	102.161 (1.802)	107.997 (3.253)	105.521 (2.991)	101.041 (1.997)	94.242 (1.836)	0.002
Hypertension						0.75
No	147 (39.510)	37 (40.802)	37 (38.170)	44 (44.800)	29 (34.738)	
Yes	303 (60.490)	76 (59.198)	75 (61.830)	69 (55.200)	83 (65.262)	
Smoke (%)						0.259
Former	164 (38.976)	47 (44.155)	41 (38.592)	36 (37.571)	40 (35.695)	
Never	223 (50.422)	63 (54.746)	53 (49.027)	53 (45.645)	54 (52.074)	
Now	63 (10.602)	3 (1.100)	18 (12.381)	24 (16.785)	18 (12.232)	
Alcohol user (%)						0.346
Former	111 (21.918)	28 (19.973)	23 (21.046)	27 (18.737)	33 (27.584)	
Heavy	41 (8.148)	7 (2.780)	7 (2.654)	15 (17.337)	12 (10.316)	
Mild	186 (45.028)	48 (48.255)	49 (47.993)	50 (44.392)	39 (39.560)	
Moderate	53 (13.658)	17 (16.702)	17 (16.918)	6 (8.646)	13 (12.103)	
Never	59 (11.247)	13 (12.290)	16 (11.389)	15 (10.888)	15 (10.437)	
Hyperlipidemia (%)						0.786
No	80 (20.231)	19 (17.448)	20 (20.126)	23 (25.612)	18 (18.044)	
Yes	370 (79.769)	94 (82.552)	92 (79.874)	90 (74.388)	94 (81.956)	
CKD						0.001
No	340 (78.711)	102 (91.711)	85 (80.576)	81 (79.791)	72 (63.171)	
Yes	110 (21.289)	12 (8.289)	27 (19.424)	30 (20.209)	41 (36.829)	

NLR, neutrophil-to-lymphocyte ratio; DRT, delayed recall test; IRT, immediate recall test; CERAD W-L, Consortium to Establish a Registry for Alzheimer’s Disease Word Learning subtest; DSST, Digit Symbol Substitution test; AFT, Animal Fluency test; CF, cognitive function; PHQ-9, Patient Health Questionnaire-9; BMI, body mass index; PIR, poverty-income ratio.

The analysis revealed that participants with higher NfL levels exhibited lower scores in DRT, DSST, AFT, total-CF, history of CKD. They also displayed higher age and serum creatinine (Table 1, *p* < 0.05). Participants with higher NfL levels exhibited no significant differences in smoking status, alcohol use, ethnicity, PIR, hyperlipidemia, education, sex, neutrophil-to-lymphocyte ratio, neutrophil proportion, lymphocyte proportion, uric acid, IRT, and hypertension.

### Association between NfL and low total-CF

Table 2 presents the associations between NfL and various indicators of low CF. After adjusting for potential confounding factors, the odds ratios (ORs) with 95% confidence intervals (CIs) for low CF indicate a significant association between NfL and an increased risk of DSST and total CF, while no significant correlation was observed between NfL and AFT and CERAD W-L.

**Table 2:** Weighted odds ratios (95% confidence intervals) of low CF by continuous and quartiles of NFL.

	*N*	TOATL-CF			AFT			DSST			CERAD W-L		
		Crude model	Model 1	Model 2	Crude model	Model 1	Model 2	Crude model	Model 1	Model 2	Crude model	Model 1	Model 2
		OR 95%CI	95%CI	95%CI	95%CI	95%CI	95%CI	95%CI	95%CI	95%CI	95%CI	95%CI	95%CI
NFL		1.034 (1.020,1.049)*	1.035 (1.017,1.053)*	1.028 (1.014,1.042)*	1.02 (1.00,1.04)	1.01 (0.98,1.04)	1.01 (0.99,1.03)	1.032 (1.018,1.047)*	1.037 (1.011,1.064)*	1.026 (1.003, 1.050)*	1.015 (1.001,1.028)*	1.010 (0.995,1.026)	1.006 (0.991, 1.020)
Q1	114	ref	ref	ref	ref	ref	ref	ref	ref	ref	ref	ref	ref
Q2	112	1.515 (0.832, 2.762)	1.455 (0.563, 3.758)	1.362 (0.720,2.576)	0.77 (0.30,2.02)	0.57 (0.16,2.06)	0.47 (0.16,1.39)	2.549 (1.108, 5.864)*	2.380 (1.013,5.596)*	3.006 (1.528, 5.914)*	1.294 (0.593,2.826)	1.114 (0.475,2.612)	1.035 (0.398, 2.691)
Q3	111	1.857 (1.050, 3.284)*	1.245 (0.441, 3.515)	1.320 (0.435,4.005)	1.92 (0.70,5.27)	1.17 (0.36,3.75)	1.19 (0.41,3.41)	2.353 (1.127, 4.913)*	2.005 (0.926,4.341)	1.813 (0.446, 7.374)	1.772 (0.767,4.098)	1.392 (0.551,3.517)	1.409 (0.535, 3.712)
Q4	113	4.151 (1.691,10.194)*	4.067 (1.445,11.451)*	3.573 (1.289,9.901)*	1.44 (0.57,3.64)	0.85 (0.30,2.41)	0.66 (0.33,1.34)	4.114 (1.662,10.184)*	3.370 (1.294,8.780)*	3.437 (1.111,10.635)*	2.622 (1.228,5.601)*	1.963 (0.806,4.782)	1.823 (0.684, 4.861)
*p* trend		0.005*	0.027*	0.038*	0.14	0.78	0.81	0.002*	0.012*	0.152	0.004*	0.055	0.093

*p < 0.05. Crude model: nfl. Model 1: nfl, sex, age, education, ethnicity. Model 2: nfl, age, sex, education, Hypertension, ethnicity, alcohol user, PHQ-9 score, poverty, CKD. NFL, Neurofilament Light Chain; CERAD W-L, Consortium to Establish a Registry for Alzheimer’s Disease Word Learning subtest; DSST, Digit Symbol Substitution test; AFT, Animal Fluency test; CF, cognitive function; *N*, sample size; CKD, Chronic Kidney Disease; PHQ-9, Patient Health Questionnaire-9.

NfL was analyzed both as a continuous and a categorized variable (four groups), as shown in Table 2. In the continuous model (per 1 unit), following multivariate adjustments (model 2), the results for total CF showed an OR of 1.028 (95%CI = 1.015–1.041 *p* < 0.001), while the results for DSST indicated an OR of 1.026 (95%CI = 1.003–1.050, *p* = 0.027).

As a categorized variable, we also computed the odds ratios (ORs) for the risk of total CF in each quartile of NfL, with Q1 as the reference group. After multivariate adjustments (model 2), the OR (95% CI) for low CF in DSST was 3.437 (95%CI = 1.111–10.635, *p* = 0.034) for the highest quartile compared to the lowest quartile of NfL. Similarly, for total CF, the OR (95% CI) for low CF was 3.573 (95%CI = 1.289–9.901, *p* = 0.018) for the highest quartile compared to the lowest quartile of NfL.

**Figure 2:**
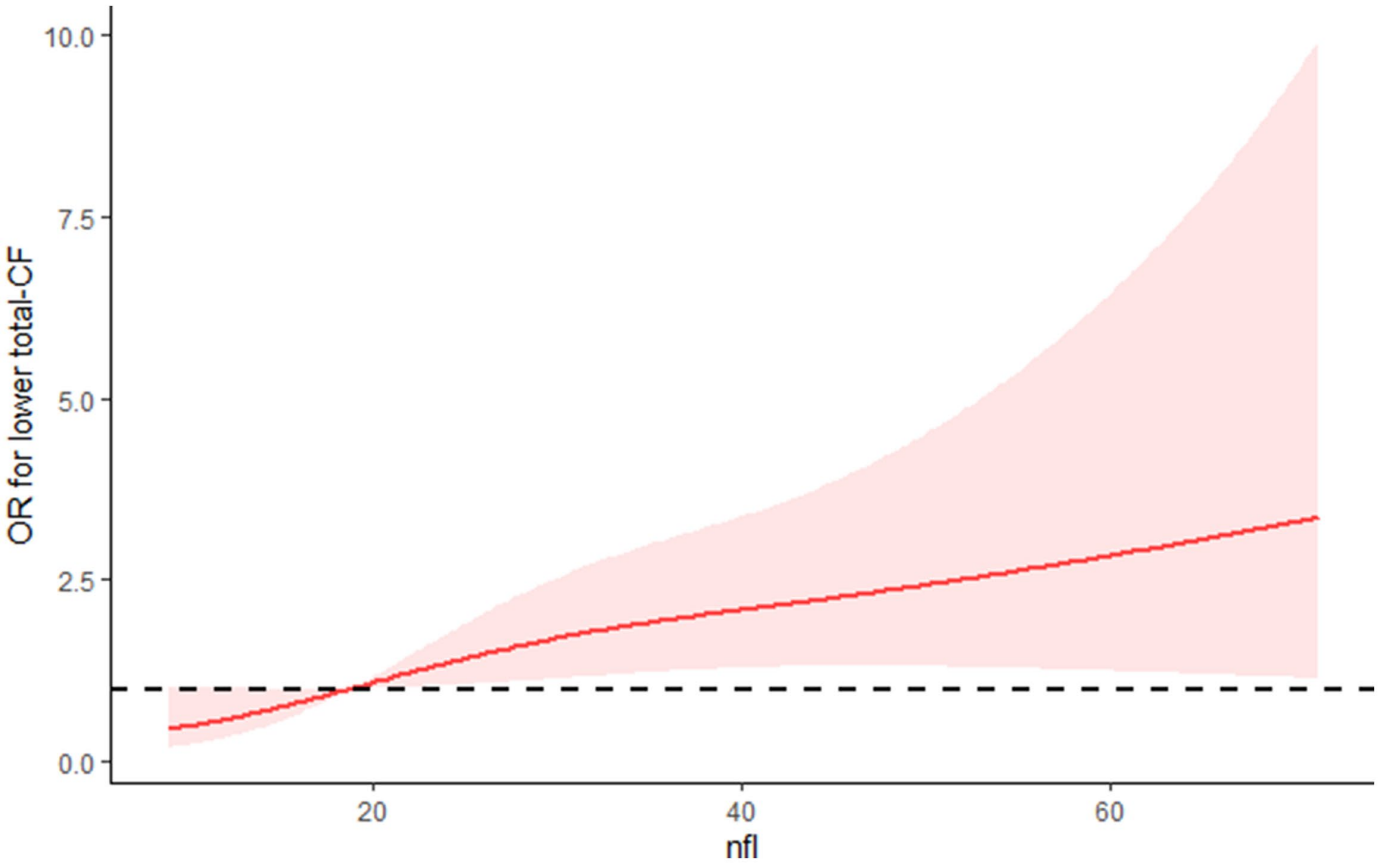
The RCS curve of the association between NFL and low total-CF among all the study participants.

Using the RCS model, it was observed that the relationship between NfL and low total-CF displayed a linear (Figure 2).

### Stratification analyses

Stratification analyses found consistent results across different variables, such as age, sex, BMI, smoking status, history of hyperlipidemia, and history of hypertension (all *p* interactions >0.05, Supplementary Figure S2).

### Identification of key low CF-related factors

**Figure 3:**
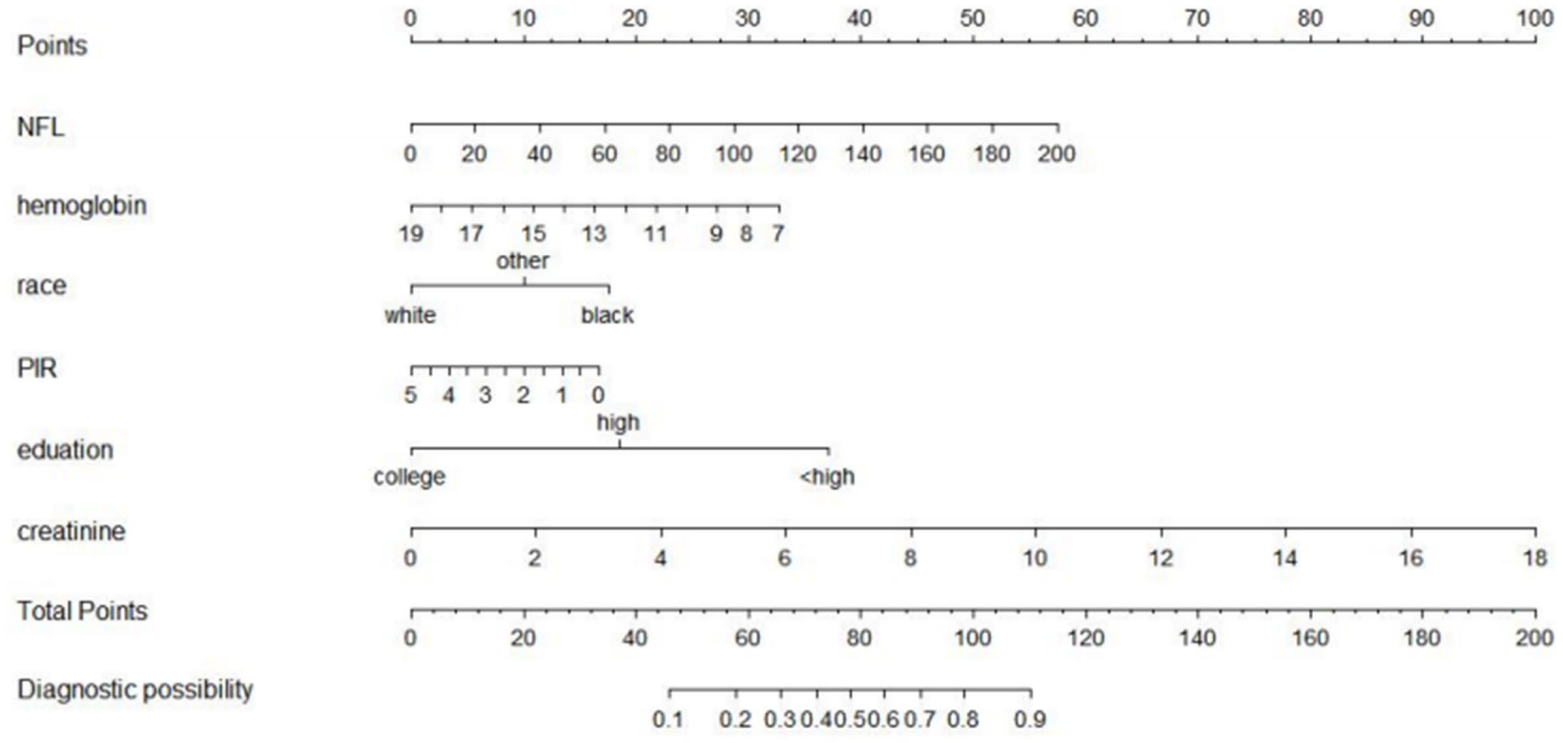
A nomogram model based risk factors identified by LASSO regression analysis. Predictive nomogram model for low total-CF in AGING people. The nomogram model was based on gender, height, and waist circumference and each predictive factor has a scoring point, the total score points of those six factors may indicate the risk of low total-CF in AGING people.

**Figure 4:**
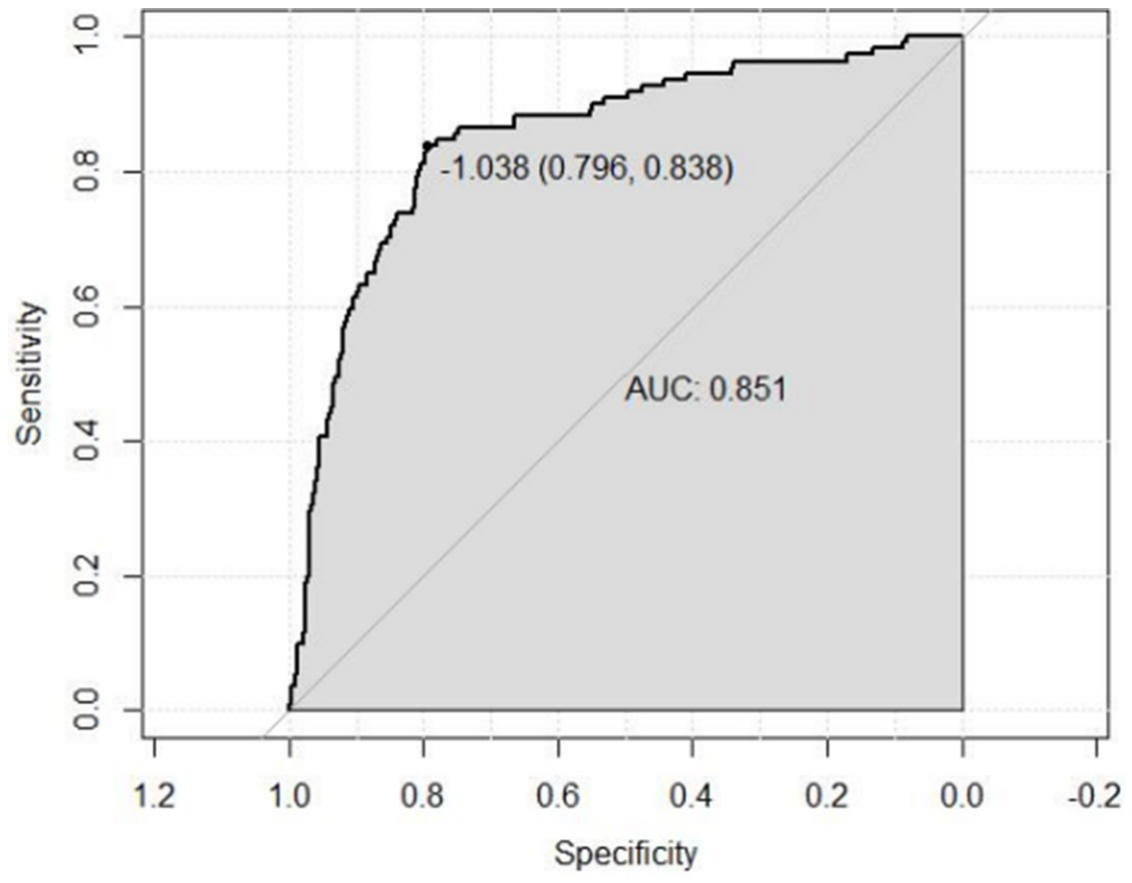
ROC curve for evaluating the predictive power for low total-CF of the nomogram model. Receiver operating characteristic curve of the nomogram. The area under the receiver operating characteristic curve (AUC) in the training set was 0.851.

**Figure 5:**
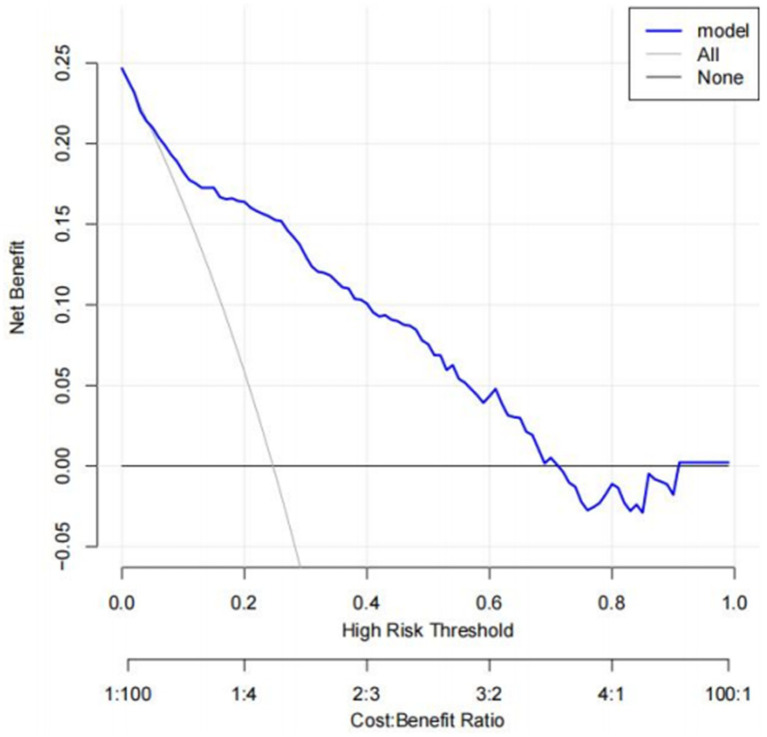
Decision curve analysis (DCA). The net benefit curves for the nomogram model are shown. *X*-axis indicates the threshold probability for low CF risk and *Y*-axis indicates the net benefit. The blue line represents the improvement predictive nomogram. The gray line represents the assumption that all patients used the nomogram model. The black line represents the assumption that no patients use the nomogram model to predict the risk of low CF.

**Figure 6:**
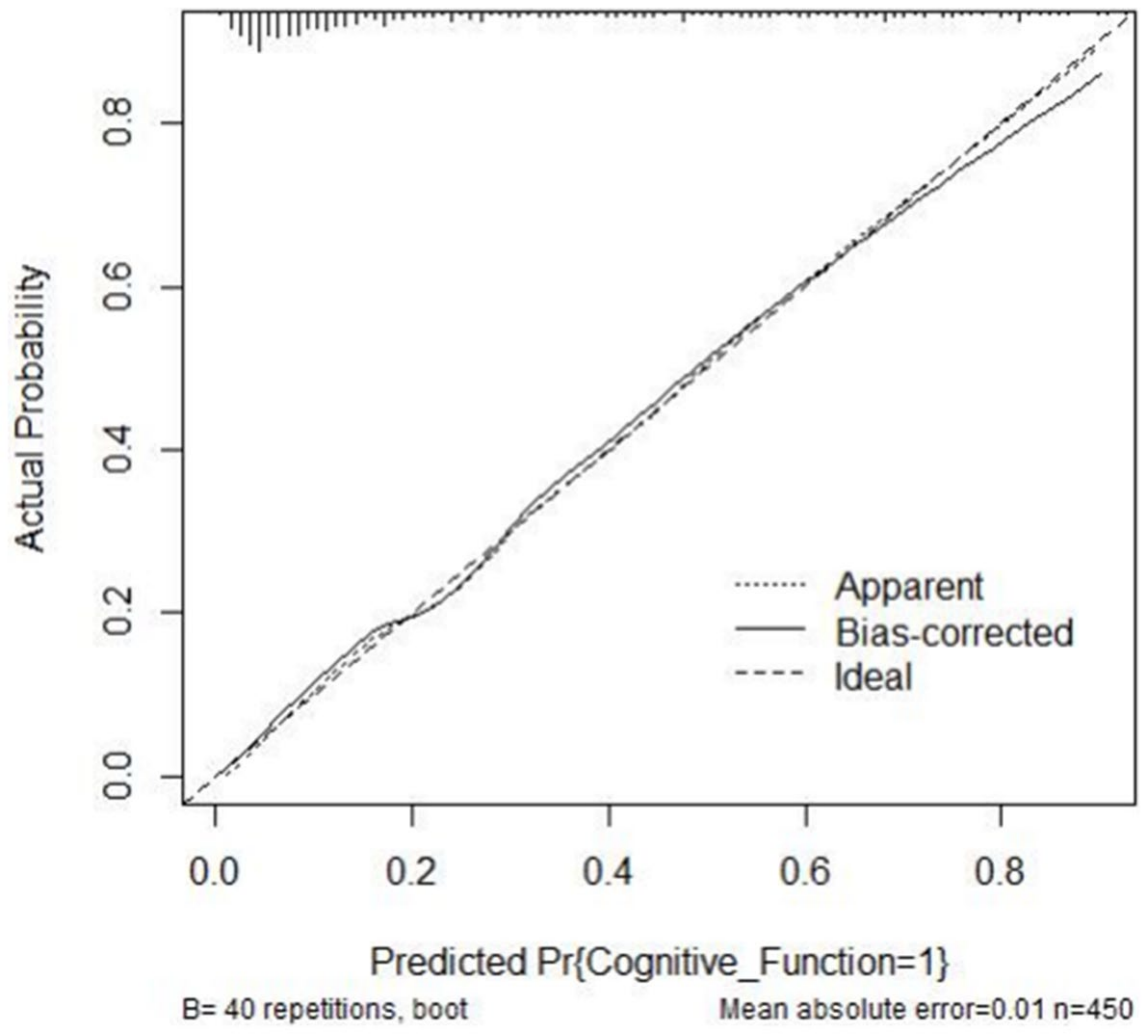
Calibration curve of the nomogram. The actual outcome rate is plotted on the y-axis; the nomogram-predicted probability of the outcome is plotted on the x-axis.

A risk prediction model based on LASSO regression, was created to identify risk factors that possessed the closest relations with low-CF (Supplementary Figures S1, S3). We conducted a Variance Inflation Factor (VIF) analysis to assess the extent of multicollinearity. To establish a risk prediction nomogram model, a total of 6 variables that NfL, race, education, hemoglobin, and serum creatinine, and PIR were selected as the risk factors most intimately correlated with low-CF were initially included (Figure 3). Its considerable predictive performance for low-CF being validated by ROC curve [AUC = 85.1% (81.6–89.3%)] (Figure 4). The decision curves showed that the model had a net benefit when the risk threshold was between 0.05 and 0.7 (Figure 5). A calibration curve also indicated good consistency between the prediction and observed outcomes (Figure 6).

## Discussion

This study investigates the relationship between serum NfL levels and cognitive function. We found a strong association between NfL levels and overall cognitive performance, particularly with the DSST. The RCS model further confirmed a linear relationship between NFL and total-CF. A predictive nomogram incorporated NfL and other risk factors. The nomogram demonstrated good discriminative, calibration capabilities, and clinical utility.

NfL levels can reflect changes in white matter and subcortical axonal neurodegeneration (Jonsson et al., 2012). Enn Hye Lee and colleagues found that plasma NfL levels are negatively correlated with cortical thickness in the parietal, temporal, and occipital lobes, as well as with the average cortical thickness across the entire brain (Lee et al., 2022). This could partly reflect the rate of cognitive decline and cortical thinning (Preische et al., 2019). Furthermore, NfL levels can also reflect MRI volume loss in regions associated with frontotemporal dementia, and may even have higher sensitivity than MRI itself (Giannini et al., 2023). These findings suggest that elevated serum NfL levels could increase the risk of brain atrophy, slowed brain metabolism, and progression to cognitive impairment stages (Mattsson et al., 2017). However, there is controversy in the existing research findings. Some studies with mixed samples have reported a negative correlation between high plasma NfL levels and low MMSE scores (Sun et al., 2022; Xiao et al., 2021), while Mielke, Pereira, and others found no significant association between blood NfL levels and cognition (Mielke et al., 2019; Pereira et al., 2021). This may be due to heterogeneity in different study populations and variations across cognitive domains. The standardized cognitive assessments used in this study (CERAD W-L, AFT, DSST) revealed a significant negative correlation between serum NfL levels and performance on memory and language tasks (IRT, AFT), as well as attention and processing speed (DSST), with all results being statistically significant (*p* < 0.05). Additionally, when summarizing the DSST, AFT, and CERAD W-L, we found that NfL was also negatively correlated with total CF. Clinically, the evaluation of overall cognitive function typically involves assessing an individual’s performance across multiple cognitive domains, such as attention, memory, language, executive function, and visuospatial ability, which collectively reflect the brain’s overall capacity for information processing. Therefore, elevated plasma NfL levels may serve as an early warning sign of cognitive decline, providing a basis for further screening and diagnosis of the disease.

Previous research by Akamine et al. found a positive correlation between blood NfL levels and serum creatinine levels, suggesting that blood NfL levels may be partially influenced by renal function (Akamine et al., 2020). Furthermore, our results also indicate a negative correlation between serum NfL levels and renal function, underscoring the significance of chronic kidney disease in older adults on NfL levels (Coresh et al., 2003). To further validate the relationship between plasma NfL levels and total CF, we used an RCS model for analysis. The results suggested a linear relationship between NfL and total CF. Additionally, a NfL-related nomogram model was developed using LASSO regression, which included seven risk factors in the predictive model: NfL, age, race, education, hemoglobin, serum creatinine, and PIR. Primary care physicians or clinicians can use the established simplified nomogram to preliminarily assess the risk stratification of a patient’s overall CF. As is well known, objective cognitive and functional impairments are evaluated through neuropsychological assessments, which typically require multiple dimensions for quantification and evaluation. In this study, by incorporating biomarkers into the model, we used the nomogram approach to assess total CF. This approach provides predictive value for overall cognitive function and can aid in the early diagnosis, screening, and treatment interventions of the disease.

There are also some limitations to the current study. First, the cross-sectional nature of the study makes it challenging to establish a causal relationship between NfL and CF. Second, the cognitive tests from NHANES data did not cover all domains of cognitive function. Definitive conclusions about general cognitive functioning require a thorough evaluation of various abilities. Third, the study population included participants > 60 years of age; thus, the results cannot be generalized to the general population. Fourth, limited to the sample size of the database, this may lead to decreased statistical power of the prediction model and cause bias of the results.

## Conclusion

In summary, serum NfL levels are closely associated with the progression of cognitive function disorders. When compared to variables such as age and sex, plasma NfL emerges as the most reliable predictive indicator for cognitive decline, especially in patients with cognitive impairment resulting from nonneurological conditions. In the early stages of the disease, the combination of NfL with cognitive function assessments proves valuable for screening and aiding in the diagnosis of cognitive function, making it a valuable biological marker. However, due to the multitude of factors contributing to cognitive decline and the complexity of other influencing factors in the body, plasma NfL lacks strong specificity and cannot further differentiate between various types of dementia. In clinical practice, it is necessary to combine it with other auxiliary tests for a comprehensive assessment and analysis.

## Data Availability

The original contributions presented in the study are included in the article/Supplementary material, further inquiries can be directed to the corresponding authors.

## Footnotes

^1^ wwwn.cdc.gov/Nchs/Data/Nhanes/Public/2013/DataFiles/SSSNFL_H.htm#SSNFLL
