# Supplementary figures and images for: Association of serum neurofilament light chain with cognitive impairment: findings from the National Health and Nutrition Examination Survey
Source: Front Aging Neurosci. 2025 Jan 20;17:1517663. doi: 10.3389/fnagi.2025.1517663 (PMC11788381; doi:10.3389/fnagi.2025.1517663)

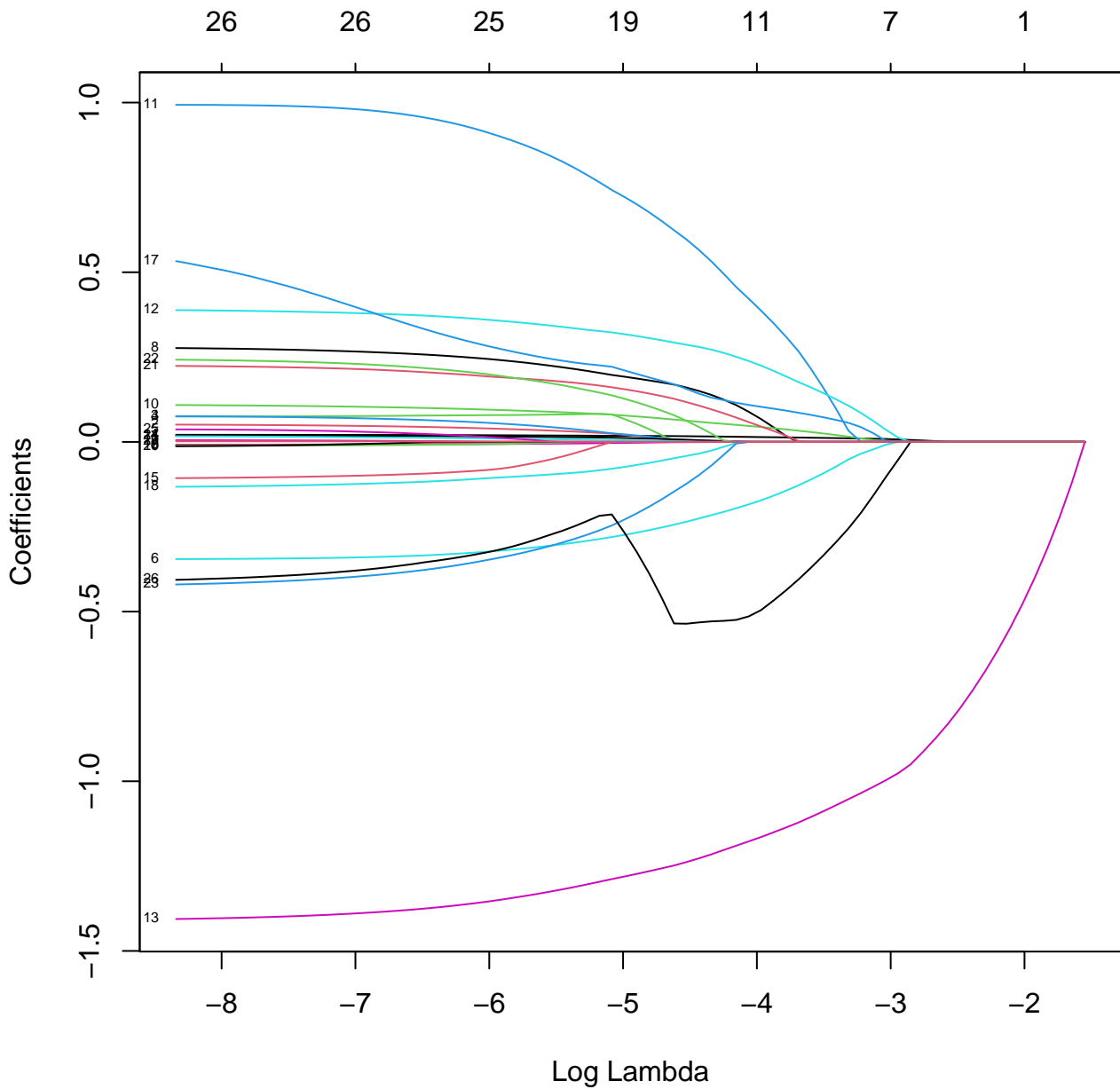

Supplement: Supplementary file 1 [file Data_Sheet_1.PDF]

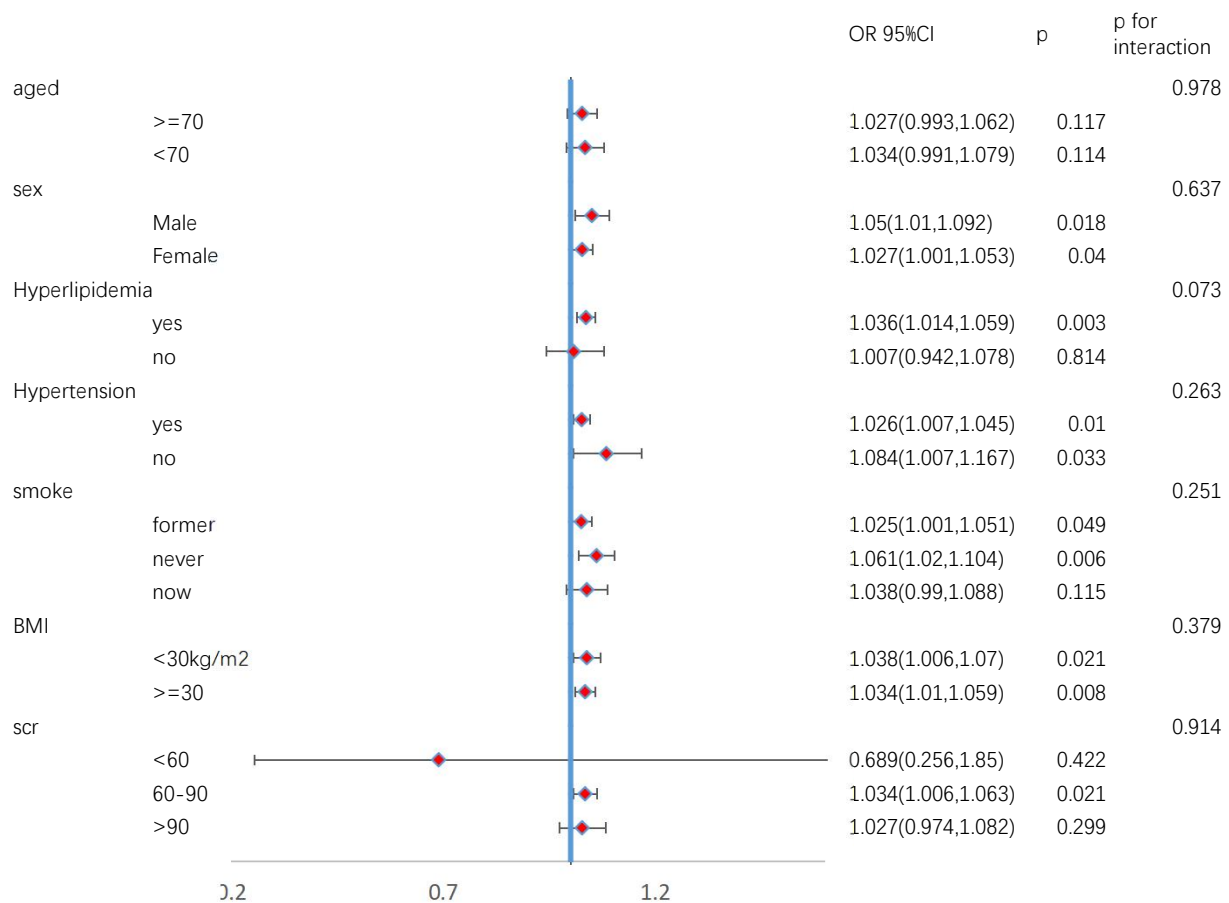

Supplement: Supplementary file 2 [file Data_Sheet_2.PDF]

Binomial Deviance

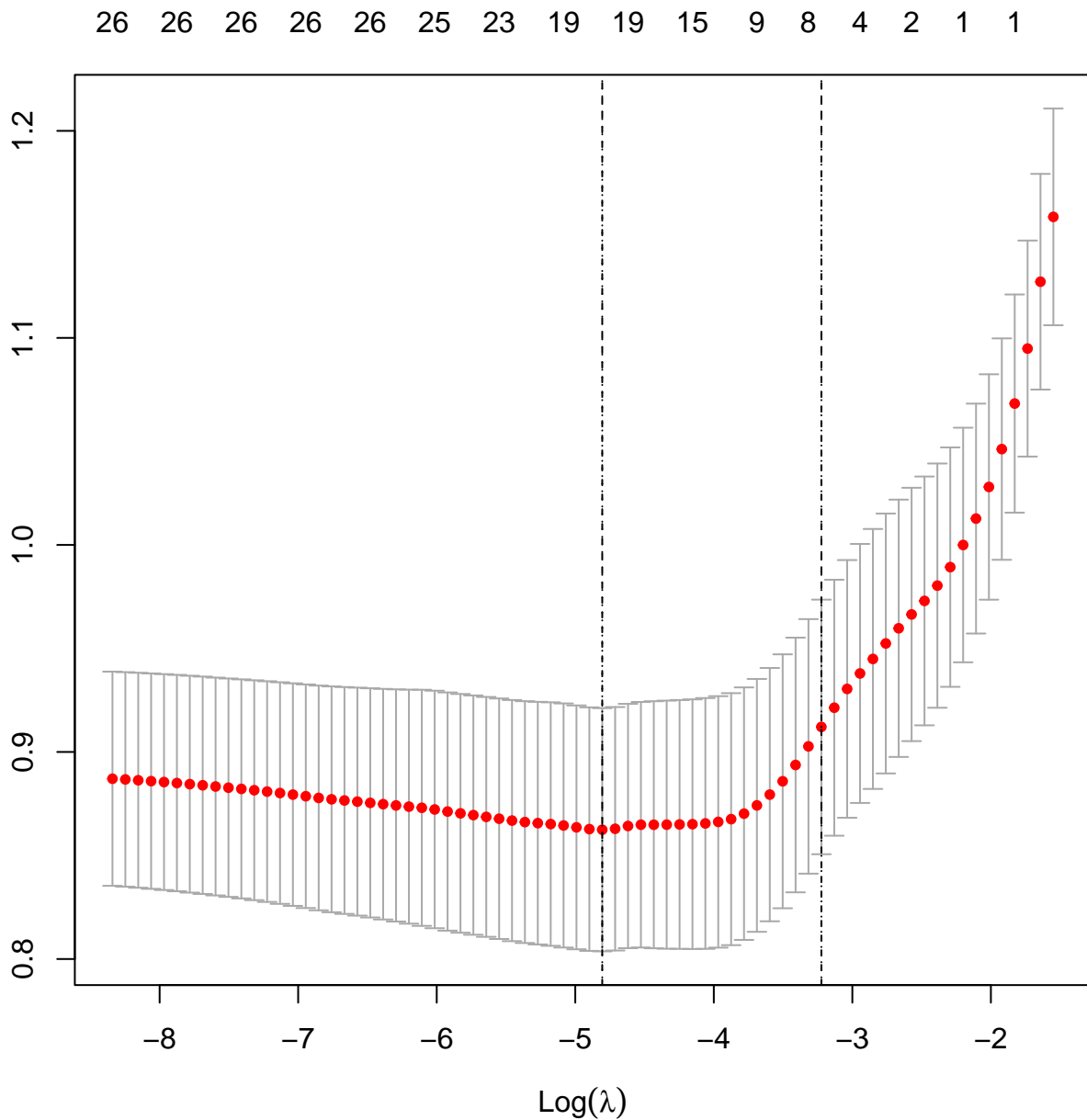

Supplement: Supplementary file 3 [file Data_Sheet_3.PDF]
